# Prognostic Significance of Visit‐to‐visit Blood Pressure Variability for Cognitive Outcomes, Vascular Events, and All‐Cause Mortality in a Memory‐Clinic Cohort

**DOI:** 10.1002/alz.095086

**Published:** 2025-01-09

**Authors:** Jiangbo Cui, Saima Hilal, Christopher Chen

**Affiliations:** ^1^ Yong Loo Lin School of Medicine, National University of Singapore, Singapore Singapore; ^2^ Memory, Ageing and Cognition Centre, National University Health System, Singapore Singapore; ^3^ Saw Swee Hock School of Public Health, National University of Singapore and National University Health System, Singapore, Singapore Singapore

## Abstract

**Background:**

The clinical relevance of visit‐to‐visit blood pressure variability (BPV) remains unclear in the memory‐clinic setting. We aimed to investigate the prognostic significance of BPV for cognitive outcomes, vascular events, and all‐cause mortality in a memory‐clinic population.

**Method:**

The study included 590 participants from an ongoing longitudinal memory clinic study who had ≥ 3 annual visits and a baseline MRI scan. Cognitive decline was defined as an annual increase of 0.5, 1, and 2 in the Clinical Dementia Rating sum of boxes for participants with No Cognitive impairment (NCI), Cognitive Impairment No Dementia (CIND), and dementia, respectively. Incident dementia was identified as transition from NCI or CIND to dementia. Incident vascular event was characterized as occurrence of cardiovascular disease, transient ischemic attack, or stroke. Significant cerebrovascular disease (sCeVD) was defined by MRI visual grading, and medial temporal lobe atrophy score ≥2 indicated neurodegeneration. Time‐dependent joint models were used, adjusting for mean BP, sCeVD, neurodegeneration, and other confounding factors, and accounting for mortality as a competing risk when necessary.

**Result:**

Over a mean (SD) follow‐up of 3.28 (1.12) years after the second BP measurement, 294 cases of cognitive decline, 75 cases of incident dementia, 34 vascular events, and 59 cases of all‐cause mortality were recorded. Per 1‐SD increase in systolic BPV (SBPV) is associated with a higher risk of cognitive decline (HR: 1.03, 95% CI: 1.00 to 1.05, p = 0.021) and a higher risk of dementia (HR: 1.09, 95% CI: 1.02 to 1.15, p = 0.002). Subgroup analyses showed that association of SBPV with cognitive decline was significant in dementia‐free participants but non‐significant in those with dementia. These associations remained significant after adjusting for sCeVD and neurodegeneration. Increased SBPV was also associated with all‐cause mortality (HR: 1.08, 95% CI: 1.03 to 1.13, p = 0.006), but not with vascular events. Similar findings were observed with diastolic BPV.

**Conclusion:**

Increased visit‐to‐visit BPV was associated with cognitive decline independent of mean BP, sCeVD and neurodegeneration among non‐demented older adults. Our findings suggest BPV may be a modifiable risk factor for adverse events and provide prognostic insights beyond mean BP in memory clinics.

